# Association between STAT4 gene polymorphism and type 2 diabetes risk in Chinese Han population

**DOI:** 10.1186/s12920-021-01000-2

**Published:** 2021-06-27

**Authors:** Jiaqi Cui, Rui Tong, Jing Xu, Yanni Tian, Juan Pan, Ning Wang, Huan Chen, Yanqi Peng, Sijia Fei, Wang Ling, Chaoying Guo, Juanchuan Yao, Wei Cui

**Affiliations:** 1 Department of Endocrinology and Second Department of Geriatrics, The First Affiliated Hospital of Xi’an Jiaotong University, #277 West Yanta Road, Xi’an, 710061 Shaanxi China; 2 Department of Oncology, East Branch of The First Affiliated Hospital of Xi’an Jiaotong University, Xi’an, 710089 Shaanxi China; 3 Department of Endocrinology, Xianyang Central Hospital, Xianyang, 712000 Shaanxi China

**Keywords:** Diabetes, *STAT4*, Single nucleotide polymorphism, Chinese Han population

## Abstract

**Background:**

Evidence from genetic epidemiology indicates that type 2 diabetes (T2D) has a strong genetic basis. Activated *STAT4* has an inflammatory effect, and STAT4 is an important mediator of inflammation in diabetes. Our study aimed to study the association between STAT4 single nucleotide polymorphisms (SNPs) and T2D susceptibility in Chinese Han population.

**Methods:**

We conducted a 'case–control' study among 500 T2D patients and 501 healthy individuals. 5 candidate *STAT4* SNPs were successfully genotyped. The association between SNPs and T2D susceptibility under different genetic models was evaluated by logistic regression analysis. ‘SNP-SNP’ interaction was analyzed and completed by multi-factor dimensionality reduction (MDR). Finally, we evaluated the differences of clinical characteristics under different genotypes by one-factor analysis of variance.

**Results:**

The overall results showed that *STAT4* rs3821236 was associated with increasing T2D risk under allele (OR 1.23, *p* = 0.020), homozygous (OR 1.51, *p* = 0.025), dominant (OR 1.36, *p* = 0.029), and additive models (OR 1.23, *p* = 0.020). The results of stratified analysis showed that rs3821236, rs11893432, and rs11889341 were risk factors for T2D among participants ≤ 60 years old. Only rs11893432 was associated with increased T2D risk among female participants. There was also a potential association between rs3821236 and T2D with nephropathy risk. STAT4 rs11893432, rs7574865 and rs897200 were significantly associated with lysophosphatidic acid, cystatin C and thyroxine t4, respectively.

**Conclusion:**

The genetic polymorphisms of STAT4 is potentially associated with T2D susceptibility of Chinese population. In particular, rs3821236 is significantly associated with T2D risk both in the overall and several subgroup analyses. Our study may provide new ideas for T2D individualized diagnosis/protection.

**Supplementary Information:**

The online version contains supplementary material available at 10.1186/s12920-021-01000-2.

## Background

Diabetes is a disease of various metabolic disorders caused by impaired glucose metabolism characterized by hyperglycemia [1, 2]. The study found that with the gradual passage of time, the age of onset of diabetes tends to be younger [3]. Its incidence rate has increased year by year and diabetes has become an important public health problem globally. At present, China has become the second largest country in the world after India in terms of number of diabetic patients. It is estimated that the total number of diabetes patients in China will be close to 100 million by 2025 [4]. According to previous reports, it is generally believed that diabetes is often caused by the interaction of genetic and environmental factors resulting in insufficient insulin secretion. Evidence from genetic epidemiology indicates that the onset of type 2 diabetes has a strong genetic basis, and its genetic model belonged to polygenetics [5]. In recent years, with the development of molecular biology and molecular epidemiology and the improvement and application of gene detection technology, some genetic polymorphism loci associated with type 2 diabetes have been identified [6]. Up to now, T2D risk assessments have been conducted only in some populations. Therefore, it is still a difficult task to discover genetic polymorphism loci associated with T2D risk-among populations with different genetic backgrounds.

STAT4 is expressed in immunoregulatory cells such as monocytes, dendritic cells, and macrophages at the site of inflammation. STAT4 mainly induces Th1 responses and inhibits Th2responses [7, 8]. Activated STAT4 is considered to have inflammatory effect, it plays an important role in the regulation of Th1/Th2 differentiation and the autoimmune diseases caused by this disorder. STAT4 is an important mediator of inflammation in immune cells and fat cells in diabetes and obesity [9]. More importantly, several studies have found Th1/Th2 cytokine imbalance in T2D patients [10–12], we speculate that STAT4 gene may play a potential role in the occurrence and development of type 2 diabetes. STAT4 genetic polymorphisms associated with the development of various diseases have been reported [13–19]. We did not find any reports on the association between STAT4 genetic polymorphisms and T2D risk.

Therefore, this study took the Chinese Han population as the research object and selected 5 candidate STAT4 SNPs (rs3821236 A/T, rs11893432 G/C, rs11889341 T/C, rs7574865 T/G and rs897200 C/T). Finally, we evaluated the association between STAT4 SNPs and T2D susceptibility. Our study may provide supplementary data for T2D risk assessment of specific population, and may also provide valuable reference for T2D individualized prevention.

## Methods

### Study objects and sample collection

After we fully obtained the consent of all participants, a total of 1001 Chinese Han people participated in this study (500 T2D patients and 501 healthy individuals with age and gender matched). Based on the genotyping results of all participants, we mainly used GCTA software (GCTA 1.26.0) to perform principal component analysis (PCA) and construct a kinship matrix to evaluate the genetic relationship between participants in this study [20]. The specific operations are as follows: (1) Plink software (PLINK v1.90b6.12) was used to convert the file format of genotyping data, which is necessary for PCA construction through GCTA software. When performing PCA, we set pca = 4. Then we used R software (R4.0.3) to draw a scatter plot based on the file generated by GCTA. Finally, the genetic relationship between the participants was estimated according to the scatter plot. (2) We used the Plink software to convert the file format of the genotyping data. The GCTA software was used to calculate the genetic relationship matrix (GRM). Finally, the kinship matrix heat map was drawn using R software, and the kinship relationship between participants was estimated according to the kinship coefficient.

#### Case group

The 500 diabetic patients come from the First Affiliated Hospital of Xi’an Jiaotong university. Among them, 142 female, accounting for 28.4%; 358 male, accounting for 71.6%. T2D inclusion criteria are as follows: (1) outpatients or inpatients of the First Affiliated Hospital of Xi'an Jiaotong University; (2) patients who have been clearly diagnosed as T2D or newly diagnosed patients with T2D (diagnostic criteria: fasting blood glucose ≥ 7.0 mmol/L/OGTT 2 h blood glucose ≥ 11.1 mmol/L/random blood glucose ≥ 11.1 mmol/L); (3) the T2D patients have no history of major mental trauma, and no history of genetic diseases: such as history of malignant tumors. All research subjects gave informed consents.

#### Control group

The 501 controls were healthy individuals selected at the same time and place as the above case group. Among them, 143 were female, accounting for 28.5%; 358 were male, accounting for 71.5%. The controls were selected according to the following requirements: (1) healthy individuals undergoing physical examination in the same hospital outpatient department at the same time; (2) fasting venous plasma glucose value ≤ 6.1 mmol/L; (3) healthy individuals without complicated chronic diseases and surgical diseases, and tumor patients or people with tumor history are excluded; (4) the basic information (age and gender) of healthy individuals is not significantly different from the case group (excluding the difference in the distribution of exposure factors between case/control caused by confounding factors).

This study was conducted under the standard approved by the First Affiliated Hospital of Xi’an Jiaotong University. All participants took part in a questionnaire about demographic and anthropological information, such as: gender, height, weight, smoking, drinking, systolic blood pressure (SBP), diastolic blood pressure (DBP), and family history of diabetes etc.

### Sample collection

We used vacuum blood collection tubes containing ethylenediaminetetraacetic acid (EDTA) to collect the fasting venous blood about 2 ml of all participants in the morning, then placed it in a refrigerator at − 20 °C to be stored until use.

#### DNA extraction

The whole genome DNA purification kit (GoldMag Co. Ltd. Xi’an, China) was used for this study, the specific experimental steps were shown in Additional file 1. The DNA was stored in the refrigerator at − 80 °C until use.

#### Selection of SNPs

The selection of SNPs should follow the principle that the allele frequency of this locus is ≥ 5% in the study population. We also calculated the successful genotyping rate (call rate) of each candidate SNPs, then filtered out the SNPs with call rate < 90%. Eliminating low-quality loci will help improve the reliability of the analysis results and reduce the false positive rate. According to the relevant literature and the data of *STAT4* gene polymorphism in the database, we finally selected 5 sites of *STAT4* gene for research (rs3821236 A/T, rs11893432 G/C, rs11889341 T/C, rs7574865 T/G and rs897200 C/T).

#### Genotyping

We use MassARRAY Assay Design software for primer design. And weused the MassARRAY system (Agena, San Diego, CA, USA) to genotype all SNPs.The MassARRAY platform is based on the MALDI-TOF (Matrix-Assisted Laser Desorption/Ionization-Time of Flight) mass spectrometer, which has the characteristics of high throughput and cost-effectiveness. The iPLEX chemical method was used to generate SNP genotypes. The specific experimental steps are as follows: (1) The region targeted by multiplex analysis is amplified by PCR (catalog number 10500). (2) The PCR product is treated with shrimp alkaline phosphatase (SAP) to neutralize unincorporated nucleotides (Cat. No. #08040). (3) Then perform an extension reaction to extend the PCR fragment by one base to the SNP site (catalog number 10136). (4) Then use MALDI-TOF to measure the quality of the obtained extension fragments to obtain the spectra of different mass peaks used for multiple reactions. Eventually we will successfully complete the genotyping.

#### Quality control

In order to verify the repeatability of the experiment, 10% of the DNA samples were randomly selected for repeated testing, and the agreement rate of the experimental results was > 99%.

### Statistical analyses

In this study, SPSS 17.0 statistical packages [21] was used to detect whether the SNPs of *STAT4* conformed to Hardy–Weinberg equilibrium (HWE). After testing whether all candidate SNPs meet Hardy–Weinberg balance, the differences in the demographic characteristics (such as: age, gender, whether smoking, drinking, and BMI) of participants in this study were tested by the chi-square test/t-test (t test was used for continuous variables such as age, and whether the mean value has statistical difference between the case group and the control group; the chi-square test was used for categorical variables such as gender, and whether the frequency distribution was statistically different between the case group and the control group). The *p* value represents whether the result is statistically significant. The logistic regression model (Adjusted by gender and age) was used to analyze and calculate the odds ratio (OR) and 95% confidence interval (CI) to evaluate the association between *STAT4* polymorphism and type 2 diabetes risk. The reason why the logistic regression analysis only adjusted by age and gender is because the age and gender data of all participants are complete (There were large missing data on ‘BMI, drinking, smoking’), which will effectively remove the influence of confounding factors on the accuracy of the results. The value of OR represents the odds ratio. When OR 1, it means that the factor has no effect on the occurrence of the disease; when OR > 1, it is a risk factor; when OR < 1, it is a protective factor.

Using wild-type alleles as reference, SNPstats online tool software was used to estimate multiple genetic models (codominant, dominant, recessive, and log-additive models). We used multifactor dimensionality reduction (MDR) to assess ‘SNP-SNP’ interaction in diabetes risk. I We used one-way analysis of variance to assess the differences in clinical indicators between different genotypes (SPSS 17.0 statistical packages). All tests are two-sided tests, and *p* < 0.05 is considered statistically significant.

## Result

### Sample introduction and collection

A total of 1,001 unrelated Chinese Han people participated in this study. We chose the ‘case–control’ experiment design type. The case group included 500 diabetic patients with an average age of 59.87 ± 12.87 years, the control group included 501 healthy individuals with an average age of 59.85 ± 9.34 years. It can be seen that there was no statistical difference in gender and age between the case group and the control group (Table 1). In addition, there was no statistical difference in smoking history or BMI between the control group and the case group, but the p-values were both closed to 0.05. And there was a very significant difference in drinking history. Contributing to the above results might be the lack of sample data. The results of principal component analysis (Additional file 1: Fig. 1) and the kinship matrix heat map (Additional file 2: Fig. 2) can be seen that all participants can be considered to have no genetic relationship. The fasting blood glucose and urea content in the diabetes case group was higher than that in the control group, while the total cholesterol content was lower in the case group than in the control group. And the above indicators showed a significant difference between the two groups (*p* < 0.001), with statistical significance. The specific data information is summarized in Table 1.

**Table 1:** The demographic and clinical characteristics of diabetic patients and controls

Characteristics	Controls	Cases	*p* value
	(n = 501)	(n = 500)	
*Age, years* (mean ± SD)	59.85 ± 9.34	59.87 ± 12.87	0.973
> 60 years old	268 (53%)	240 (48%)	
≤ 60 years old	233 (47%)	260 (52%)	
*Gender*			0.960
Male	358(71%)	358(72%)	
Female	143(29%)	142(28%)	
*Drinking*			** < 0.001**
Yes	103 (21%)	109 (22%)	
No	140 (28%)	385 (77%)	
*Smoking*			0.085
Yes	98 (20%)	219 (44%)	
No	164 (33%)	280 (56%)	
*BMI*			0.062
> 24	130 (26%)	239 (48%)	
≤ 24	188 (38%)	203 (41%)	
*FPG (mmol/L)*			** < 0.001**
Mean ± SD	6.05 ± 1.60	7.35 ± 3.40	
Number	386 (77%)	455 (91%)	
*Creatinine (mg/dL)*			0.371
Mean ± SD	68.74 ± 12.87	71.20 ± 52.66	
Number	385 (77%)	485 (97%)	
*ALT (IU/L)*			0.133
Mean ± SD	27.66 ± 31.35	24.75 ± 25.87	
Number	385 (77%)	492 (98%)	
*TBA*			0.299
Mean ± SD	6.66 ± 18.22	5.70 ± 5.29	
Number	385 (77%)	421 (84%)	
*Urea*			** < 0.001**
Mean ± SD	5.42 ± 2.78	6.52 ± 3.26	
Number	384 (77%)	484 (97%)	
*TC*			** < 0.001**
Mean ± SD	4.93 ± 4.00	4.18 ± 2.01	
Number	385 (77%)	496 (99%)	

SD: standard deviation;

BMI: body mass index;

FPG: fasting plasma glucose;

ALT: alanine transaminase;

TBA: total bile acids;

TC: total cholesterol

**Fig. 1:**
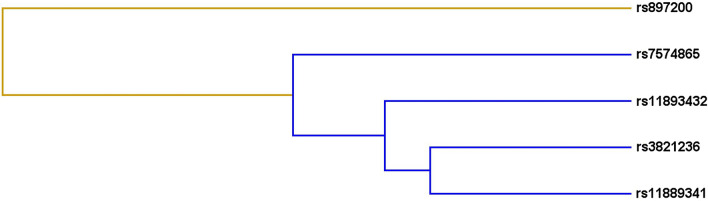
Multifactor dimensionality reduction (MDR) analysis of STAT4 rs3821236, rs11893432, rs11889341, rs7574865 and rs897200 interaction. The colors in the tree diagram represent synergy (yellow) or redundancy (blue)

### Association between STAT4 polymorphism and type 2 diabetes risk

In this study, a total of 5 SNPs (rs3821236, rs11893432, rs11889341, rs7574865 and rs897200) were successfully genotyped. The call rate of all loci was more than 90% (Table 2), which will help to improve the reliability of the results. The detailed information of candidate SNPs is listed in Table 2. All candidate SNPs are in HWE (*p* > 0.05). And the minor allele frequency (MAF) of all candidate SNPs are greater than 5% in the test population. The analysis results of HaploReg show that 5 SNPs are regulated by various factors, such as promoter histone marks, enhancer histone marks, motifs changed, NHGRI/EBI GWAS hits, GRASP QTL hits, Selected eQTL hits, etc. This study used logistic regression (Adjusted by gender and age) to test the association between SNPs and diabetes risk under different genetic models.

**Table 2:** The basic information of *STAT4* polymorphisms

Gene	SNP ID	Chr:Position	Alleles (A/B)	Call rate	MAF		HWE (*p* Value)	Haploreg 4.1	SNPinfo web serve
					Cases	Controls			
*STAT4*	rs3821236	Chr2: 191902758	A/G	100%	0.479	0.427	0.784	Promoter histone marks; Enhancer histone marks; Motifs changed; NHGRI/EBI GWAS hits; GRASP QTL hits; Selected eQTL hits	
*STAT4*	rs11893432	Chr2: 191921874	G/C	100%	0.483	0.441	0.928	Enhancer histone marks; Motifs changed; Selected eQTL hits	
*STAT4*	rs11889341	Chr2: 191943742	T/C	99.3%	0.355	0.330	0.545	Promoter histone marks; Enhancer histone marks; Motifs changed; NHGRI/EBI GWAS hits;	
*STAT4*	rs7574865	Chr2: 191964633	T/G	100%	0.347	0.327	0.223	Enhancer histone marks; Motifs changed; NHGRI/EBI GWAS hits; GRASP QTL hits;	
*STAT4*	rs897200	Chr2: 192017771	C/T	100%	0.489	0.494	0.475	Enhancer histone marks; DNAse; Proteins bound; Motifs changed; NHGRI/EBI GWAS hits; Selected eQTL hits	TFBS

*HWE* Hardy–Weinberg equilibrium, *SNP* single nucleotide polymorphisms, *MAF* minor allele frequency

*p* > 0.05 indicates that the genotypes were in Hard-Weinberg Equilibrium;

#### Overall analysis

Comprehensive analysis of all data, the result showed (Table 3) that among the 5 candidate SNPs in this study, only the rs3821236 polymorphism was associated with T2D risk, and the remaining four were not been found to be significantly associated with T2D risk (*p* > 0.05). Specifically, the results of this study showed that the genotype frequencies of rs3821236 (AA, AG and GG) in the case group were 22.6%, 50.6%, and 26.8%, while in the control group were 18.6%, 48.3%, and 33.1%, respectively. Among them, the allele (A vs. G, OR 1.23, CI 1.03–1.47, *p* = 0.020) and homozygous (AA vs. GG, OR 1.51, CI 1.05–2.15, *p* = 0.025) models were positively associated with increased risk of T2D. At the same time, we found that the rs3821236 polymorphism had a significant association with the increased risk of diabetes under dominant (GG vs. AA-AG, OR 1.36, CI 1.03–1.78, *p* = 0.029) and log-additive models (OR 1.23, CI 1.03–1.47, *p* = 0.020).

**Table 3:** Analysis of the association between diabetes risk and single nucleotide polymorphism of *STAT4*

SNP ID	Model	Genotype	Case	Control	Adjusted by age and gender	
					OR (95% CI)	*p*
rs3821236	Allele	G	521	574	1.00	
		A	479	428	1.23 (1.03–1.47)	0.020*
	Genotype	AA	113	93	1.51 (1.05–2.15)	0.025*
		AG	253	242	1.30 (0.97–1.73)	0.077
		GG	134	166	1.00	
	Dominant	AA-AG	366	335	1.36 (1.03–1.78)	0.029*
		GG	134	166	1.00	
	Recessive	AA	113	93	1.28 (0.94–1.74)	0.114
		AG-GG	387	408	1.00	
	Log-additive	–	–	–	1.23 (1.03–1.47)	0.020*
rs11893432	Allele	C	517	560	1.00	
		G	483	442	1.18 (0.99–1.41)	0.060
	Genotype	GG	116	98	1.40 (0.98–1.99)	0.064
		GC	251	246	1.21 (0.90–1.61)	0.208
		CC	133	157	1.00	
	Dominant	GG-GC	367	344	1.26 (0.96–1.66)	0.098
		CC	133	157	1.00	
	Recessive	GG	116	98	1.24 (0.92–1.68)	0.161
		GC-CC	384	403	1.00	
	Log-additive	–	–	–	1.18 (0.99–1.41)	0.060
rs11889341	Allele	C	645	669	1.00	
		T	355	329	1.12 (0.93–1.35)	0.233
	Genotype	TT	53	51	1.16 (0.75–1.78)	0.499
		TC	249	227	1.22 (0.94–1.59)	0.131
		CC	198	221	1.00	
	Dominant	TT-TC	302	278	1.21 (0.94–1.56)	0.134
		CC	198	221	1.00	
	Recessive	TT	53	51	1.04 (0.69–1.56)	0.845
		TC-CC	447	448	1.00	
	Log-additive	–	–	–	1.13 (0.93–1.36)	0.220
rs7574865	Allele	G	653	673	1.00	
		T	347	327	1.09 (0.91–1.32)	0.344
	Genotype	TT	49	47	1.14 (0.73–1.77)	0.571
		TG	249	233	1.17 (0.90–1.51)	0.253
		GG	202	220	1.00	
	Dominant	TT-TG	298	280	1.16 (0.90–1.49)	0.247
		GG	202	220	1.00	
	Recessive	TT	49	47	1.05 (0.69–1.60)	0.828
		TG-GG	451	453	1.00	
	Log-additive	–	–	–	1.10 (0.91–1.34)	0.322
rs897200	Allele	T	511	507	1.00	
		C	489	495	0.98 (0.82–1.17)	0.823
	Genotype	CC	125	118	0.97 (0.68–1.37)	0.846
		CT	239	259	0.84 (0.62–1.14)	0.260
		TT	136	124	1.00	
	Dominant	CC-CT	364	377	0.88 (0.66–1.17)	0.377
		TT	136	124	1.00	
	Recessive	CC	125	118	1.08 (0.81–1.45)	0.594
		CT-TT	375	383	1.00	
	Log-additive	–	–	–	0.98 (0.82–1.17)	0.822

*CI* confidence interval, *OR* odds ratio

*p*: values were calculated by unconditional logistic regression analysis with adjustment for age and gender; *p < 0.05 indicates statistical significance

### Age and gender (Table 4)

**Table 4:** The SNPs of *STAT4* associated with diabetes risk in the subgroup tests

SNP ID	Model	genotype	Case	Control	OR (95% CI)	*p*	Case	Control	OR (95% CI)	*p*
Age, years			> 60				≤ 60			
rs3821236	Allele	G	212	232	1.00		267	196	1.00	
		A	268	304	1.04 (0.81–1.33)	0.777	253	270	1.45 (1.13–1.87)	0.004
	Genotype	GG	73	90	1.00		61	76	1.00	
		AA	45	54	0.91 (0.54–1.54)	0.729	68	39	2.16 (1.28–3.64)	0.004
		AG	122	124	1.15 (0.76–1.73)	0.507	131	118	1.39 (0.91–2.12)	0.127
	Dominant	GG	73	90	1.00		61	76	1.00	
		AA-AG	167	178	1.08 (0.73–1.58)	0.710	199	157	1.58 (1.06–2.35)	0.025
	Recessive	AG-GG	195	214	1.00		192	194	1.00	
		AA	45	54	0.84 (0.53–1.33)	0.454	68	39	1.75 (1.12–2.73)	0.014
	Log-additive	-	-	-	0.98 (0.76–1.26)	0.864	-	-	1.46 (1.13–1.90)	0.004
rs11893432	Allele	C	214	239	1.00		269	203	1.00	
		G	266	297	1.00 (0.78–1.28)	0.998	251	263	1.39 (1.08–1.79)	0.010
	Genotype	CC	74	85	1.00		59	72	1.00	
		GG	48	56	0.89 (0.53–1.49)	0.664	68	42	1.97 (1.17–3.32)	0.010
		CG	118	127	1.00 (0.66–1.51)	1.000	133	119	1.39 (0.91–2.13)	0.130
	Dominant	CC	74	85	1.00		59	72	1.00	
		GG-GC	166	183	0.97 (0.66–1.43)	0.866	201	161	1.54 (1.03–2.31)	0.036
	Recessive	GC-CC	192	212	1.00		192	191	1.00	
		GG	48	56	0.84 (0.53–1.33)	0.454	68	42	1.59 (1.03–2.46)	0.038
	Log-additive	-	-	-	0.95 (0.74–1.23)	0.695	-	-	1.40 (1.08–1.82)	0.010
rs11889341	Allele	C	158	181	1.00		197	148	1.00	
		T	322	355	0.96 (0.74–1.25)	0.774	323	314	1.29 (0.99–1.69)	0.055
	Genotype	CC	107	119	1.00		91	102	1.00	
		TT	25	32	0.87 (0.47–1.60)	0.644	28	19	1.60 (0.83–3.06)	0.161
		TC	108	117	1.01 (0.69–1.48)	0.944	141	110	1.46 (1.00–2.13)	0.052
	Dominant	CC	107	119	1.00		91	102	1.00	
		TT-TC	133	149	0.98 (0.68–1.41)	0.925	169	129	1.48 (1.03–2.13)	0.037
	Recessive	TC-CC	215	236	1.00		232	212	1.00	
		TT	25	32	0.86 (0.48–1.54)	0.611	28	19	1.29 (0.70–2.39)	0.416
	Log-additive	-	-	-	0.96 (0.73–1.26)	0.757	-	-	1.34 (1.01–1.78)	0.046
rs7574865	Allele	G	154	173	1.00		193	154	1.00	
		T	326	361	0.99 (0.76–1.28)	0.915	327	312	1.20 (0.92–1.56)	0.182
	Genotype	GG	109	121	1.00		93	99	1.00	
		TT	23	27	1.01 (0.54–1.91)	0.972	26	20	1.32 (0.68–2.53)	0.412
		TG	108	119	1.01 (0.69–1.47)	0.967	141	114	1.33 (0.91–1.94)	0.138
	Dominant	GG	109	121	1.00		93	99	1.00	
		TT-TG	131	146	1.01 (0.70–1.45)	0.963	167	134	1.33 (0.92–1.91)	0.127
	Recessive	TG-GG	217	240	1.00		234	213	1.00	
		TT	23	27	1.01 (0.55–1.85)	0.981	26	20	1.12 (0.60–2.08)	0.722
	Log-additive	-	-	-	1.01 (0.76–1.33)	0.963	-	-	1.22 (0.92–1.62)	0.177
rs897200	Allele	T	236	265	1.00		245	224	1.00	
		C	244	271	0.99 (0.77–1.27)	0.931	275	242	0.96 (0.75–1.24)	0.765
	Genotype	TT	65	69	1.00		75	58	1.00	
		CC	61	66	1.13 (0.68–1.88)	0.626	60	49	0.91 (0.54–1.52)	0.713
		CT	114	133	1.00 (0.64–1.54)	0.990	125	126	0.75 (0.49–1.15)	0.191
	Dominant	TT	65	69	1.00		75	58	1.00	
		CC-CT	175	199	1.04 (0.69–1.57)	0.847	185	175	0.80 (0.53–1.19)	0.268
	Recessive	CT-TT	179	202	1.00		200	184	1.00	
		CC	61	66	1.14 (0.75–1.72)	0.549	60	49	1.10 (0.71–1.69)	0.677
	Log-additive	-	-	-	1.06 (0.83–1.37)	0.630	-	-	0.94 (0.73–1.22)	0.648

SNP ID	Model	genotype	Case	Control	OR (95% CI)	*p*	Case	Control	OR (95% CI)	*p*
Gender			Male				Female			
rs3821236	Allele	G	336	302	1.00		143	126	1.00	
		A	380	414	1.21 (0.98–1.49)	0.071	141	160	1.29 (0.93–1.79)	0.154
	Genotype	GG	101	122	1.00		33	44	1.00	
		AA	79	66	1.45 (0.95–2.20)	0.085	34	27	1.69 (0.85–3.33)	0.132
		AG	178	170	1.27 (0.90–1.77)	0.172	75	72	1.39 (0.80–2.40)	0.245
	Dominant	GG	101	122	1.00		33	44	1.00	
		AA-AG	257	236	1.32 (0.96–1.81)	0.090	109	99	1.47 (0.87–2.49)	0.153
	Recessive	AG-GG	279	292	1.00		108	116	1.00	
		AA	79	66	1.25 (0.87–1.81)	0.227	34	27	1.36 (0.77–2.40)	0.295
	Log-additive	-	-	-	1.21 (0.98–1.49)	0.073	-	-	1.30 (0.93–1.83)	0.125-
rs11893432	Allele	C	338	322	1.00		145	120	1.00	
		G	378	394	1.09 (0.89–1.35)	0.396	139	166	1.44 (1.04–2.01)	0.029
	Genotype	CC	100	111	1.00		33	46	1.00	
		GG	80	75	1.18 (0.78–1.79)	0.425	36	23	2.19 (1.10–4.37)	0.026
		GC	178	172	1.15 (0.82–1.62)	0.427	73	74	1.38 (0.79–2.40)	0.254
	Dominant	CC	100	111	1.00		33	46	1.00	
		GG-GC	258	247	1.16 (0.84–1.60)	0.367	109	97	1.57 (0.93–2.66)	0.092
	Recessive	GC-CC	278	283	1.00		106	120	1.00	
		GG	80	75	1.09 (0.76–1.55)	0.650	36	23	1.78 (0.99–3.19)	0.054
	Log-additive	-	-	-	1.09 (0.89–1.34)	0.400	-	-	1.47 (1.05–2.07)	0.027
rs11889341	Allele	C	257	236	1.00		98	93	1.00	
		T	459	476	1.13 (0.91–1.41)	0.275	186	193	1.09 (0.77–1.55)	0.615
	Genotype	CC	140	157	1.00		58	64	1.00	
		TT	39	37	1.18 (0.71–1.96)	0.516	14	14	1.11 (0.49–2.52)	0.810
		TC	179	162	1.24 (0.91–1.69)	0.178	70	65	1.19 (0.73–1.94)	0.489
	Dominant	CC	140	157	1.00		58	64	1.00	
		TT-TC	218	199	1.23 (0.91–1.66)	0.176	84	79	1.18 (0.73–1.88)	0.502
	Recessive	TC-CC	319	319	1.00		128	129	1.00	
		TT	39	37	1.06 (0.66–1.70)	0.827	14	14	1.01 (0.46–2.21)	0.981
	Log-additive	-	-	-	1.14 (0.91–1.43)	0.261	-	-	1.10 (0.77–1.58)	0.599
rs7574865	Allele	G	252	233	1.00		95	94	1.00	
		T	464	483	1.13 (0.90–1.40)	0.289	189	190	1.02 (0.72–1.44)	0.929
	Genotype	GG	142	158	1.00		60	62	1.00	
		TT	36	33	1.26 (0.72–2.05)	0.467	13	14	0.96 (0.42–2.23)	0.933
		TG	180	167	1.20 (0.88–1.64)	0.249	69	66	1.08 (0.66–1.77)	0.752
	Dominant	GG	142	158	1.00		60	62	1.00	
		TT-TG	216	200	1.20 (0.89–1.62)	0.225	82	80	1.06 (0.66–1.70)	0.802
	Recessive	TG-GG	322	325	1.00		13	14	1.00	
		TT	36	33	1.10 (0.67–1.81)	0.704	129	128	0.92 (0.42–2.05)	0.848
	Log-additive	-	-	-	1.14 (0.91–1.43)	0.268	-	-	1.02 (0.71–1.47)	0.916
rs897200	Allele	T	346	350	1.00		141	141	1.00	
		C	370	366	0.98 (0.79–1.20)	0.833	143	145	1.01 (0.73–1.41)	0.934
	Genotype	TT	102	91	1.00		35	35	1.00	
		CC	90	83	0.97 (0.64–1.46)	0.875	34	33	1.03 (0.53–2.01)	0.930
		CT	166	184	0.80 (0.57–1.14)	0.227	73	75	0.97 (0.55–1.72)	0.927
	Dominant	TT	102	91	1.00		35	35	1.00	
		CC-CT	256	267	0.86 (0.61–1.19)	0.354	107	108	0.99 (0.58–1.70)	0.974
	Recessive	CT-TT	268	275	1.00		108	110	1.00	
		CC	90	83	1.11 (0.79–1.57)	0.541	34	33	1.05 (0.61–1.81)	0.864
	Log-additive	-	-	-	0.98 (0.80–1.20)	0.833	-	-	1.02 (0.73–1.42)	0.933

*CI* confidence interval, *OR* odds ratio

*p*: values were calculated by unconditional logistic regression analysis with adjustment for age and gender, ^*^p < 0.05 indicates statistical significance

The study population was grouped according to age (60 years old as the dividing line) and gender (male and female) to analyze the association between genetic polymorphisms and T2Drisk in different subgroups. The rs3821236, rs11893432 and rs11889341 polymorphisms were positively associated with increased risk of T2D among participants aged ≤ 60 years. Specifically, rs3821236 polymorphism was associated with an increased risk of T2D in allele (A vs. G, OR 1.45, CI 1.13–1.87, *p* = 0.004), homozygous (AA vs. GG, OR 2.16, CI 1.28–3.64, *p* = 0.004), dominant (GG vs. AA-AG, OR 1.58, CI 1.06–2.35, *p* = 0.025), recessive (AG-GG vs. AA, OR 1.75, CI 1.12–2.73, *p* = 0.014), and log-additive models(OR 1.46, CI 1.13–1.90, *p* = 0.004). We also found that rs11893432 was positively associated with the risk of T2Din allele (C vs. G, OR 1.39, CI 1.08–1.79, *p* = 0.010), homozygous (CC vs. GG, OR 1.97, CI 1.17–3.32, *p* = 0.010), dominant (CC vs. GG-GC, OR 1.54, CI 1.03–2.31, *p* = 0.036), recessive (GC-CC vs. GG, OR 1.59, CI 1.03–2.46, *p* = 0.038) and log-additive models (OR 1.40, CI 1.08–1.82, *p* = 0.010). However, we only observed an association between rs11889341 polymorphism and the increased risk of T2Din dominant (CC vs. TT-TC, OR 1.48, CI 1.03–2.13, *p* = 0.037) and log-additive models (OR 1.34, CI 1.01–1.78, *p* = 0.046). Conversely, among the participants over 60 years old, there was no association between the five candidate SNPs and the T2D risk. When the study population was divided by gender to analyze, the result showed that only rs11893432 was associated with the increased risk of T2D risk among female participants: rs11893432 was a risk factor for T2D in the allele (C vs. G, OR 1.44, CI 1.04–2.01, *p* = 0.029), homozygous (CC vs. GG, OR 2.19, CI 1.10–4.37, *p* = 0.026) and log-additive models (OR 1.47, CI 1.05–2.07, *p* = 0.027).

### BMI (Table 5)

**Table 5:** The SNPs of *STAT4* associated with T2D risk in the subgroup tests (BMI)

SNP ID	Model	Genotype	OR (95% CI)	*p*	OR (95% CI)	*p*
			≤ 24		> 24	
rs3821236	Allele	A	1.30(0.95–1.79)	0.099	1.1(0.84–1.44)	0.498
		G	1.00		1.00	
	Genotype	AA	1.82(0.94–3.53)	0.076	1.18(0.69–2.04)	0.544
		AG	1.35(0.81–2.24)	0.250	1.17(0.73–1.87)	0.505
		GG	1.00		1.00	
	Dominant	AA-AG	1.46(0.90–2.36)	0.123	1.18(0.76–1.82)	0.470
		GG	1.00		1.00	
	Recessive	AA	1.51(0.85–2.71)	0.164	1.07(0.68–1.68)	0.778
		AG-GG	1.00		1.00	
	Log-additive	–	1.35(0.98–1.87)	0.070	1.09(0.83–1.43)	0.538
rs11893432	Allele	G	1.34(0.98–1.84)	0.066	1.03(0.79–1.35)	0.830
		C	1.00		1.00	
	Genotype	GG	1.83(0.95–3.52)	0.070	1.07(0.62–1.84)	0.815
		GC	1.51(0.91–2.52)	0.113	0.88(0.55–1.42)	0.609
		CC	1.00		1.00	
	Dominant	GG-GC	1.59(0.98–2.58)	0.059	0.94(0.60–1.47)	0.792
		CC	1.00		1.00	
	Recessive	GG	1.42(0.80–2.51)	0.236	1.16(0.74–1.81)	0.521
		GC-CC	1.00		1.00	
	Log-additive	–	1.37(0.99–1.90)	0.055	1.03(0.79–1.36)	0.818
rs11889341	Allele	T	1.41(1.01–1.98)	0.115	1.86(0.65–2.14)	0.282
		C	1.00		1.00	
	Genotype	TT	1.62(0.72–3.63)	0.243	1.73(1.38–2.41)	0.353
		TC	1.63(1.02–2.62)	0.143	1.29(0.52–2.21)	0.279
		CC	1.00		1.00	
	Dominant	TT-TC	1.63(1.04–2.56)	0.035*	1.78(0.52–2.17)	0.230
		CC	1.00		1.00	
	Recessive	TT	1.27(0.58–2.75)	0.549	1.84(0.45–2.54)	0.564
		TC-CC	1.00		1.00	
	Log-additive	–	1.41(0.99–2.00)	0.059	1.84(0.62–2.33)	0.239
rs7574865	Allele	T	1.36(0.97–1.91)	0.073	1.37(0.66–2.16)	0.342
		G	1.00		1.00	
	Genotype	TT	1.26(0.58–2.74)	0.555	1.48(0.39–2.15)	0.483
		TG	1.75(1.09–2.83)	0.021*	1.80(0.53–2.21)	0.288
		GG	1.00		1.00	
	Dominant	TT-TG	1.65(1.05–2.59)	0.030*	1.28(0.53–1.99)	0.263
		GG	1.00		1.00	
	Recessive	TT	0.96(0.46–2.03)	0.921	1.19(0.47–1.89)	0.721
		TG-GG	1.00		1.00	
	Log-additive	–	1.33(0.94–1.89)	0.108	1.65(0.63–2.26)	0.306
rs897200	Allele	C	0.83(0.60–1.13)	0.229	1.16(0.89–1.52)	0.274
		T	1.00		1.00	
	Genotype	CC	0.66(0.34–1.27)	0.214	1.33(0.78–2.26)	0.294
		CT	1.20(0.71–2.03)	0.503	0.86(0.53–1.39)	0.538
		TT	1.00		1.00	
	Dominant	CC-CT	1.02(0.62–1.68)	0.944	1.02(0.65–1.59)	0.933
		TT	1.00		1.00	
	Recessive	CC	0.59(0.34–1.02)	0.059	1.46(0.95–2.25)	0.085
		CT-TT	1.00		1.00	
	Log-additive	–	0.84(0.60–1.16)	0.287	1.16(0.89–1.51)	0.266

*BMI* body mass index, *CI* confidence interval, *OR* odds ratio

*p*: values were calculated by unconditional logistic regression analysis with adjustment for age and gender, ^*^p < 0.05 indicates statistical significance

The subjects were grouped according to ‘body mass index’ to analyze the association between candidate SNPs and T2D risk. The results showed that STAT4 rs11889341 (Dominant: OR 1.63, *p* = 0.035) and rs7574865 (Heterozygote: OR 1.75,* p* = 0.021; Dominant: OR 1.65, *p* = 0.030) significantly increased T2D risk in participants with BMI ≤ 24. In participants with BMI > 24, we did not find any evidence associated with T2D risk. In spite of this, the T2D risk of participants with BMI > 24 in our study almost all showed an increasing trend.

### Smoking and drinking (Table 6)

**Table 6:** The SNPs of *STAT4* associated with T2D risk in the subgroup tests (smoking and drinking status)

SNP ID	Model	Genotype	Smoking				Drinking			
			OR (95% CI)	*p*	OR (95% CI)	*p*	OR (95% CI)	*p*	OR (95% CI)	*p*
			Yes		No		Yes		No	
rs3821236	Allele	A	1.31(0.93–1.84)	0.119	1.27(0.96–1.67)	0.092	1.29(0.68–2.45)	0.964	1.42(1.07–1.87)	0.014*
		G	1.00		1.00		1.00		1.00	
	Genotype	AA	1.98(0.93–4.2)	0.076	1.53(0.88–2.65)	0.132	1.07(0.48–2.38)	0.866	1.87(1.07–3.27)	0.027*
		AG	1.08(0.62–1.86)	0.788	1.42(0.90–2.22)	0.129	1.14(0.39–2.39)	0.344	1.73(1.12–2.69)	0.014*
		GG	1.00		1.00		1.00		1.00	
	Dominant	AA-AG	1.25(0.74–2.11)	0.396	1.45(0.95–2.21)	0.084	1.02(0.45–1.89)	0.510	1.77(1.17–2.68)	0.006*
		GG	1.00		1.00		1.00		1.00	
	Recessive	AA	1.89(0.97–3.68)	0.063	1.24(0.76–2.00)	0.388	1.30(0.65–2.59)	0.460	1.35(0.82–2.21)	0.241
		AG-GG	1.00		1.00		1.00		1.00	
	Log-additive	–	1.34(0.94–1.91)	0.102	1.25(0.95–1.65)	0.108	1.00(0.67–1.48)	0.990	1.42(1.07–1.88)	0.015*
rs11893432	Allele	G	1.12(0.80–1.57)	0.511	1.26(0.96–1.66)	0.097	1.28(0.55–2.18)	0.261	1.43(1.09–1.89)	0.011*
		C	1.00		1.00		1.00		1.00	
	Genotype	GG	1.25(0.63–2.51)	0.525	1.63(0.93–2.88)	0.090	1.09(0.33–1.88)	0.347	2.03(1.15–3.61)	0.015*
		GC	1.08(0.62–1.90)	0.777	1.16(0.74–1.83)	0.510	1.16(0.32–2.13)	0.114	1.52(0.98–2.37)	0.062
		CC	1.00		1.00		1.00		1.00	
	Dominant	GG-GC	1.13(0.67–1.92)	0.649	1.29(0.84–1.97)	0.250	1.23(0.35–2.14)	0.126	1.65(1.09–2.51)	0.019*
		CC	1.00		1.00		1.00		1.00	
	Recessive	GG	1.19(0.66–2.16)	0.568	1.48(0.91–2.42)	0.115	1.05(0.50–1.92)	0.882	1.56(0.94–2.59)	0.086
		GC-CC	1.00		1.00		1.00		1.00	
	Log-additive	–	1.12(0.79–1.58)	0.531	1.27(0.96–1.67)	0.097	1.12(0.56–1.99)	0.289	1.44(1.08–1.91)	0.012*
rs11889341	Allele	T	1.17(0.82–1.67)	0.383	1.09(0.81–1.45)	0.581	0.98(0.65–1.46)	0.906	1.22(0.91–1.64)	0.184
		C	1.00		1.00		1.00		1.00	
	Genotype	TT	1.37(0.56–3.36)	0.486	1.13(0.59–2.18)	0.713	1.27(0.49–3.31)	0.621	1.09(0.57–2.08)	0.802
		TC	1.24(0.75–2.06)	0.403	1.18(0.78–1.79)	0.429	1.07(0.43–1.97)	0.371	1.61(1.06–2.44)	0.025*
		CC	1.00		1.00		1.00		1.00	
	Dominant	TT-TC	1.26(0.77–2.06)	0.354	1.17(0.79–1.74)	0.430	1.22(0.48–2.16)	0.534	1.49(1.01–2.21)	0.036*
		CC	1.00		1.00		1.00		1.00	
	Recessive	TT	1.22(0.52–2.84)	0.652	1.04(0.56–1.93)	0.905	1.47(0.59–3.62)	0.406	0.85(0.46–1.58)	0.615
		TC-CC	1.00		1.00		1.00		1.00	
	Log-additive	–	1.20(0.82–1.77)	0.354	1.10(0.82–1.48)	0.519	1.08(0.65–1.89)	0.935	1.22(0.9–1.65)	0.202
rs7574865	Allele	T	1.21(0.84–1.73)	0.303	1.03(0.77–1.38)	0.832	1.06(0.64–2.23)	0.835	1.18(0.88–1.58)	0.272
		G	1.00		1.00		1.00		1.00	
	Genotype	TT	1.73(0.65–4.61)	0.275	1.07(0.50–1.87)	0.927	1.33(0.47–3.77)	0.589	0.95(0.50–1.82)	0.883
		TG	1.18(0.71–1.95)	0.520	1.20(0.79–1.81)	0.390	1.05(0.42–2.02)	0.311	1.62(1.07–2.45)	0.023*
		GG	1.00		1.00		1.00		1.00	
	Dominant	TT-TG	1.24(0.76–2.02)	0.387	1.15(0.78–1.70)	0.482	1.11(0.47–2.04)	0.451	1.46(0.99–2.16)	0.058
		GG	1.00		1.00		1.00		1.00	
	Recessive	TT	1.58(0.61–4.04)	0.344	1.08(0.47–1.95)	0.697	1.56(0.58–4.22)	0.377	0.75(0.40–1.38)	0.349
		TG-GG	1.00		1.00		1.00		1.00	
	Log-additive	–	1.25(0.84–1.85)	0.267	1.05(0.78–1.42)	0.728	0.96(0.62–1.48)	0.843	1.17(0.86–1.58)	0.314
rs897200	Allele	C	1.02(0.66–1.89)	0.641	1.23(0.94–1.62)	0.136	0.93(0.63–1.36)	0.706	1.21(0.92–1.60)	0.165
		T	1.00		1.00		1.00		1.00	
	Genotype	CC	1.04(0.43–2.02)	0.600	1.51(0.86–2.65)	0.153	1.38(0.43–2.03)	0.737	1.62(0.90–2.91)	0.111
		CT	1.06(0.43–1.86)	0.358	1.20(0.76–1.90)	0.440	1.13(0.59–2.16)	0.711	0.88(0.56–1.39)	0.578
		TT	1.00		1.00		1.00		1.00	
	Dominant	CC-CT	1.19(0.46–2.35)	0.388	1.28(0.83–1.98)	0.261	1.03(0.57–1.87)	0.924	1.04(0.67–1.62)	0.849
		TT	1.00		1.00		1.00		1.00	
	Recessive	CC	1.00(0.57–1.92)	0.973	1.34(0.83–2.16)	0.230	1.22(0.44–1.91)	0.525	1.76(1.06–2.91)	0.028*
		CT-TT	1.00		1.00		1.00		1.00	
	Log-additive	–	1.01(0.66–2.06)	0.582	1.23(0.93–1.62)	0.154	0.94(0.66–1.36)	0.750	1.23(0.93–1.62)	0.149

*CI* confidence interval, *OR* odds ratio

*p* values were calculated by unconditional logistic regression analysis with adjustment for age and gender; ^*^p < 0.05 indicates statistical significance

The results showed that when the participants were grouped according to smoking status (Yes/No) for association analysis, we did not find any statistically significant results. Except for rs897200, the STAT4 rs3821236 (Allele: OR 1.42, *p* = 0.014; Homozygote: OR 1.42, *p* = 0.014; Heterozygote: OR 1.42, *p* = 0.014; Dominant: OR 1.42, *p* = 0.014; Log-additive: OR 1.42, *p* = 0.014), rs11893432 (Allele: OR 1.43, *p* = 0.011; Homozygote: OR 2.03, *p* = 0.015; Dominant: OR 1.65, *p* = 0.019; Log-additive: OR 1.44, *p* = 0.012), rs11889341 (Heterozygote: OR 1.61, *p* = 0.025; Dominant: OR 1.49, *p* = 0.036) and rs7574865 (Heterozygote: OR 1.62, *p* = 0.023) were all significantly associated with T2D risk among non-drinking participants. Although the five candidate SNPs had no potential association with the T2D risk among drinking participants, the T2D risk among drinking participants showed an increasing trend.

### T2D complications (Table 7)

**Table 7:** Correlation between *STAT4* gene polymorphism and the occurrence of diabetes complications

SNP ID	Model	Genotype	T2D complicated with nephropathy				T2D complicated with CHD			
			DN	No DN	OR (95% CI)	*p*	Case	Control	OR (95% CI)	*p*
rs3821236	Allele	A	127	352	0.78(0.59–1.02)	0.073	127	352	1.14(0.86–1.52)	0.359
		G	165	356	1.00		125	396	1.00	
	Genotype	AA	30	83	0.65(0.37–1.14)	0.131	30	83	1.41(0.77–2.60)	0.266
		AG	67	186	0.62(0.40–0.99)	0.024*	67	186	1.35(0.81–2.25)	0.251
		GG	49	85	1.00		29	105	1.00	
	Dominant	AA-AG	97	269	0.63(0.41–0.97)	0.037*	97	269	1.37(0.84–2.23)	0.207
		GG	49	85	1.00		29	105	1.00	
	Recessive	AA	30	83	0.88(0.54–1.42)	0.589	30	83	1.16(0.70–1.91)	0.567
		AG-GG	116	271	1.00		96	291	1.00	
	Log-additive	–	–	–	0.79(0.59–1.05)	0.099	–	–	1.19(0.88–1.61)	0.252
rs11893432	Allele	G	131	352	0.82(0.63–1.08)	0.163	126	357	1.10(0.82–1.46)	0.532
		C	161	356	1.00		126	391	1.00	
	Genotype	GG	30	86	0.71(0.40–1.24)	0.227	30	86	1.29(0.70–2.35)	0.416
		GC	71	180	0.78(0.49–1.23)	0.280	66	185	1.26(0.76–2.10)	0.371
		CC	45	88	1.00		30	103	1.00	
	Dominant	GG-GC	101	266	0.75(0.49–1.17)	0.203	96	271	1.27(0.78–2.06)	0.334
		CC	45	88	1.00		30	103	1.00	
	Recessive	GG	30	86	0.83(0.51–1.35)	0.455	30	86	1.10(0.67–1.81)	0.704
		GC-CC	116	268	1.00		96	288	1.00	
	Log-additive	–	–	–	0.84(0.63–1.11)	0.212	–	–	1.14(0.84–1.53)	0.402
rs11889341	Allele	T	97	258	0.87(0.65–1.16)	0.333	81	274	0.82(0.61–1.11)	0.198
		C	195	450	1.00		171	474	1.00	
	Genotype	TT	15	38	0.81(0.41–1.62)	0.558	10	43	0.56(0.25–1.25)	0.156
		TC	67	182	0.79(0.52–1.21)	0.280	61	188	0.89(0.57–1.37)	0.589
		CC	64	134	1.00		55	143	1.00	
	Dominant	TT-TC	82	220	0.80(0.54–1.19)	0.268	71	231	0.82(0.54–1.26)	0.370
		CC	64	134	1.00		55	143	1.00	
	Recessive	TT	15	38	0.92(0.48–1.77)	0.807	10	43	0.60(0.28–1.28)	0.188
		TC-CC	131	316	1.00		116	331	1.00	
	Log-additive	–	–	–	0.86(0.63–1.17)	0.339	–	–	0.80(0.58–1.12)	0.192
rs7574865	Allele	T	96	251	0.89(0.67–1.19)	0.437	82	265	0.88(0.65–1.19)	0.405
		G	196	457	1.00		170	483	1.00	
	Genotype	TT	14	35	0.90(0.45–1.83)	0.775	9	40	0.65(0.29–1.47)	0.302
		TG	68	181	0.84(0.55–1.27)	0.410	64	185	1.04(0.67–1.61)	0.869
		GG	64	138	1.00		53	149	1.00	
	Dominant	TT-TG	82	216	0.85(0.57–1.27)	0.423	73	225	0.97(0.63–1.48)	0.882
		GG	64	138	1.00		53	149	1.00	
	Recessive	TT	14	35	0.99(0.51–1.94)	0.980	9	40	0.64(0.29–1.39)	0.259
		TG-GG	132	319	1.00		117	334	1.00	
	Log-additive	–	–	–	0.91(0.66–1.24)	0.530	–	–	0.90(0.65–1.25)	0.524
rs897200	Allele	C	147	342	1.09(0.83–1.43)	0.558	129	360	1.13(0.85–1.5)	0.400
		T	145	366	1.00		123	388	1.00	
	Genotype	CC	35	90	1.10(0.63–1.93)	0.741	35	90	1.16(0.65–2.07)	0.605
		CT	77	162	1.44(0.89–2.33)	0.139	59	180	1.05(0.63–1.75)	0.852
		TT	34	102	1.00		32	104	1.00	
	Dominant	CC-CT	112	252	1.32(0.83–2.07)	0.239	94	270	1.09(0.68–1.75)	0.725
		TT	34	102	1.00		32	104	1.00	
	Recessive	CC	35	90	0.87(0.55–1.38)	0.545	35	90	1.13(0.70–1.82)	0.617
		CT-TT	111	264	1.00		91	284	1.00	
	Log-additive	–	–	–	1.05(0.80–1.38)	0.717	–	–	1.08(0.81–1.44)	0.606

*CHD* coronary heart disease, *CI* confidence interval, *OR* odds ratio

*p* values were calculated by unconditional logistic regression analysis with adjustment for age and gender; *p < 0.05 indicates statistical significance

Finally, we grouped the case group according to whether they complicated with nephropathy or coronary heart disease (CHD) to evaluate the association between candidate SNPs and the risk of T2D complications. The results showed that (Table 7) only rs3821236 was potentially associated with the susceptibility to T2D complicated with nephropathy under heterozygous (*p* = 0.024) and dominant (*p* = 0.037) genetic models. At the same time, the results showed that the 5 candidate SNPs didn’t associated with the susceptibility of T2D complicated with CHD.

#### Differences in clinical indicators under different genotypes

Finally, we also conducted an association study between the five candidate SNPs and clinical indicators s of T2D patients. The results showed (Table 8) that the level of clinical indicators associated with the candidate SNPs in this study were cystatin C, lysophosphatidic acid, and thyroxine. Specifically, the STAT4 rs11893432 was associated with LPa (*p* = 0.021); rs7574865 was associated with CysC (*p* = 0.033); while rs897200 had been found that was associated with T4 (*p* = 0.010). And the above data are statistically significant.

### MDR analysis

Subsequently, we used MDR analysis to evaluate the SNP-SNP interaction. The interaction between these SNPs are described as Fig. 1. The blue line indicates that these 5 SNPs may have a redundancy effect in regulating the risk of diabetes. The specific information is summarized in Table 9. The results show that the best single-site model for predicting the risk of diabetes is: rs3821236 (testing accuracy = 0.515, CVC = 9/10, *p* = 0.032); the two-site model is: rs3821236, rs897200 (testing accuracy = 0.523, CVC = 5/10, *p* = 0.011); the three-site model is: rs3821236, rs11889341 and rs897200 (testing accuracy = 0.499, CVC = 5/10, p = 0.001); the four-site model is: rs3821236, rs11893432, rs11889341 and rs897200 (testing accuracy = 0.496, CVC = 8/10, *p* < 0.0001); the five-site models are: rs3821236, rs11893432, rs11889341, rs7574865 and rs897200 (testing accuracy = 0.501, CVC = 10/10, *p* < 0.0001).

**Table 8:** Analysis of the association between clinical characteristics of diabetes patients and SNP genotypes

SNP	FPG	HbA1c	TC (mmol/L)	Urea (mmol/L)	Cys C	LPa (mg/L)	T4 (ng/ml)
*rs3821236*							
AA	7.29 ± 3.83	7.35 ± 3.40	4.16 ± 1.14	6.15 ± 2.16	0.93 ± 0.30	255.50 ± 262.67	6.95 ± 1.60
AG	7.41 ± 3.38	7.94 ± 1.99	4.22 ± 2.61	6.69 ± 4.13	1.04 ± 0.56	209.67 ± 214.89	7.00 ± 1.89
GG	7.29 ± 3.03	8.08 ± 1.97	4.13 ± 1.10	6.52 ± 1.89	1.02 ± 0.33	199.87 ± 199.50	6.82 ± 1.83
*p*	0.934	0.714	0.893	0.342	0.125	0.136	0.647
*rs11893432*							
GG	7.21 ± 3.74	7.94 ± 1.92	4.19 ± 1.19	6.18 ± 2.22	0.94 ± 0.31	269.81 ± 281.32	6.91 ± 1.68
GC	7.31 ± 3.34	8.12 ± 2.15	4.18 ± 2.61	6.65 ± 4.12	1.03 ± 0.56	205.84 ± 204.67	7.02 ± 1.88
CC	7.53 ± 3.21	8.11 ± 1.99	4.17 ± 1.09	6.57 ± 1.91	1.02 ± 0.33	193.76 ± 192.91	6.83 ± 1.79
*p*	0.768	0.735	0.997	0.438	0.205	0.021*	0.657
*rs11889341*							
TT	7.69 ± 5.02	8.03 ± 1.92	4.17 ± 1.11	6.28 ± 2.38	1.00 ± 0.35	259.67 ± 257.00	6.63 ± 1.63
TC	7.17 ± 3.30	8.10 ± 2.19	4.23 ± 2.64	6.61 ± 3.96	0.98 ± 0.45	218.45 ± 232.96	6.90 ± 1.70
CC	7.48 ± 3.03	8.05 ± 1.91	4.13 ± 1.07	6.47 ± 2.36	1.05 ± 0.48	204.18 ± 198.44	7.08 ± 1.97
*p*	0.512	0.969	0.872	0.787	0.246	0.318	0.298
*rs7574865*							
TT	6.94 ± 2.80	8.07 ± 1.98	4.17 ± 1.07	6.34 ± 2.42	1.09 ± 0.83	244.93 ± 253.88	6.52 ± 1.56
TG	7.39 ± 3.78	8.13 ± 2.20	4.22 ± 2.63	6.58 ± 3.92	0.96 ± 0.30	218.35 ± 233.32	6.94 ± 1.72
GG	7.39 ± 3.01	8.00 ± 1.90	4.14 ± 1.11	6.48 ± 2.44	1.05 ± 0.49	208.48 ± 200.50	7.05 ± 1.96
*p*	0.725	0.823	0.901	0.874	0.033*	0.618	0.246
*rs897200*							
CC	7.23 ± 3.00	8.21 ± 2.09	4.09 ± 1.00	6.35 ± 2.46	1.01 ± 0.38	212.91 ± 216.65	7.26 ± 1.93
CT	7.53 ± 3.57	8.07 ± 2.09	4.10 ± 1.10	6.76 ± 4.63	0.99 ± 0.55	209.38 ± 214.61	6.99 ± 1.90
TT	7.14 ± 3.45	7.96 ± 1.98	4.41 ± 3.44	6.76 ± 4.63	1.04 ± 0.32	233.92 ± 242.07	6.55 ± 1.44
*p*	0.558	0.628	0.297	0.502	0.698	0.600	0.010*

*HbA1c* glycosylated hemoglobin, *TC* total cholesterol, *FPG* fasting plasma glucose, CysC: cystatin C;

LPa: lysophosphatidic acid;

Lpa: lipoprotein a;

T4: thyroxine;

^*^p < 0.05 indicates statistical significance

**Table 9:** SNP–SNP interaction models analyzed by the MDR method

Model	Training Bal. Acc	Testing Bal. Acc	CVC	OR (95% CI)	*p*
rs3821236	0.532	0.515	9/10	1.35(1.03–1.77)	0.032*
rs3821236, rs897200	0.542	0.523	5/10	1.38(1.08–1.77)	0.011*
rs3821236, rs11889341, rs897200	0.555	0.499	5/10	1.52(1.19–1.96)	0.001*
rs3821236, rs11893432, rs11889341, rs897200	0.572	0.496	8/10	1.73(1.35–2.22)	< 0.0001*
rs3821236, rs11893432, rs11889341, rs7574865, rs897200	0.579	0.501	10/10	1.85(1.43–2.38)	< 0.0001*

*MDR* multifactor dimensionality reduction, *Bal. Acc.* balanced accuracy, *CVC* cross–validation consistency, *OR* odds ratio,

CI: confidence interval;

*p* values were calculated using χ^2^ tests, *p < 0.05 indicates statistical significance

## Discussion

Type 2 diabetes is the result of the interaction of genetic and environmental factors. In recent years, the association between genetic polymorphisms and diseases has been the focus of attention. Studies have found that STAT4 mainly induces Th1 response and inhibits Th2 response [7, 8]. It plays an important role in the regulation of Th1/Th2 differentiation and the autoimmune diseases caused by this disorder. Multiple studies have shown that Th1/Th2 cytokine imbalance exists in T2D patients [10–12]. However, the specific mechanism of STAT4 in T2D is still unclear. Therefore, our study conducted a study on the association between STAT4 genetic polymorphisms and T2D risk in Chinese Han population. This study will supplement the data of T2D susceptibility-associated genetic loci.

Our results showed that only rs3821236 was associated with type 2 diabetes risk among the five candidate SNPs of STAT4 (rs3821236 A/G, rs11893432 G/C, rs11889341 T/C, rs7574865 T/G and rs897200 C/T). STAT4 is an important transcriptional activator. After activation, it crosses the nuclear membrane into the nucleus in the form of a homodimer, and then initiates the transcription and expression of downstream target genes [22]. Numerous studies have found that the STAT4 rs3821236 genetic polymorphism is associated with multiple disease risks, such as systemic lupus erythematosus (SLE) [23], Systemic sclerosis [24] and juvenile idiopathic arthritis [25]. etc. This study obtained similar results to previous studies: STAT4 rs3821236 was found to have a certain association with the risk of T2D in multiple genetic models (allele model, homozygous model, dominant model, etc.), whether we are performing an overall analysis or a stratified analysis.

In recent studies reported by Zhao et al. [10] and Mahlangu et al. [11], they all found that the differentiation regulation of Th1/Th2 played a certain role in T2D. And it has been found that STAT4 plays a certain role in the regulation of Th1/Th2 differentiation. Combined with the results of our study, we speculated that STAT4 rs3821236 may play a certain role in the differentiation and regulation of Th1/Th2, which may influence T2D susceptibility. However, this is only a speculation, which may need further study in larger sample size to confirm. Nevertheless, as far as we know, our study is the first to find evidence that STAT4 rs3821236 is potentially associated with the occurrence and development of T2D in Chinese Han population. It will provide new ideas for the individualized treatment or diagnosis of T2D.

On the other hand, genetic and environmental factors are interrelated in T2D and promote its development. The previous study has shown that age, obesity and unhealthy lifestyle are risk factors for T2D [26]. Therefore, this study also conducted a stratified analysis related to the above. Our results showed: among the population ≤ 60 years old, rs3821236, rs11893432 and rs11889341 of STAT4 were significantly associated with increased T2D risk; among the population with BMI < 24, rs11889341 and rs7574865were significantly associated with increased risk of T2D; among the non-drinking population, rs3821236, rs11893432, rs11889341 and rs7574865 had a certain association with the increased risk ofT2D; in the analysis of whether the participants smoked, there was no significant association between STAT4 gene polymorphism and T2D risk. The above results seemed to be inconsistent with previous studies. We were pleasantly surprised to find that although there was no significant association between STAT4 gene polymorphism and T2D susceptibility among participants with potential T2D risk, it was showed an increasing trend of T2D risks among these participants. The result indicates that STAT4 gene polymorphism is associated with increased T2D risk, which may be greatly affected by genetic factors, while the environmental factors may have little effect.

In addition, we found that there are some differences between the results of our study and previous studies: STAT4 rs7574865 gene polymorphism is a risk factor for increasing the risk of diabetes in Asians and Caucasians [27], while according to the results of this study, rs7574865 was only associated with the clinical indicator (cystatin C, *p* = 0.033). However, it is not sufficient to prove that rs7574865 is associated with T2D risk. We speculate that the causes for the above differences may be different research populations, inconsistent sample sizes and different research environments etc.

Our study provides data supplement for the study of the association between STAT4 gene polymorphism and the risk of T2D in Chinese Han population: there is a certain association between the two. However, this study still has certain limitations. Because of the small sample size and missing sample data (BMI, drinking, smoking). Only two baselines of age and gender were adjusted in the logistic regression to ensure the accuracy of the results. In subsequent studies, we need to further expand the sample size to continue the study, so as to more strongly confirm the results of our study.

## Conclusion

In summary, the study is the first study of the association between *STAT4* gene polymorphism and T2D risk in Chinese Han population. Our results suggest that *STAT4* gene polymorphism (rs3821236, rs11893432, rs11889341, rs7574865, rs897200) has a potential association with the risk of T2D in the Chinese Han population. It provides supplementary data for the in-depth study of the association between the STAT4 gene and T2D risk. And it can provide a theoretical and scientific basis for the preliminary molecular basis of prevention and treatment for T2D from a genetic perspective.

## Supplementary Information

**Additional file 1**: Figure 1 Principal component analysis based on genotyping data of 1001 participants. The distance of each sample on the horizontal and vertical axes represents the similarity distance influenced by the principal component. The stronger the relevance of participants, the closer they are in PCA; the weaker the relevance of participants, the more scattered they are in PCA.

**Additional file 2**: Figure 2 Heat map of kinship matrix for1001 participants. The color of each lattice represents the correlation between rows and columns. The more red the color is, the stronger positive correlation is; and the more blue the color is, the stronger negative correlation is.

**Additional file 3**: Table Genotyping results of all participants.

## Data Availability

The datasets supporting the conclusions of this article are included within the article and its Additional file 3.

## Abbreviations

STAT4: Signal Transcription and Transducer 4
T2D: Type 2 diabetes
SNPs: Single nucleotide polymorphisms
PCA: Principal component analysis
OR: Odds ratio
CI: Confidence interval
MDR: Multifactor dimensionality reduction
MAF: Minor allele frequency
SLE: Systemic lupus erythematosus
