# Supplementary figures and images for: Association between STAT4 gene polymorphism and type 2 diabetes risk in Chinese Han population
Source: BMC Med Genomics. 2021 Jun 27;14:169. doi: 10.1186/s12920-021-01000-2 (PMC8237503; doi:10.1186/s12920-021-01000-2)

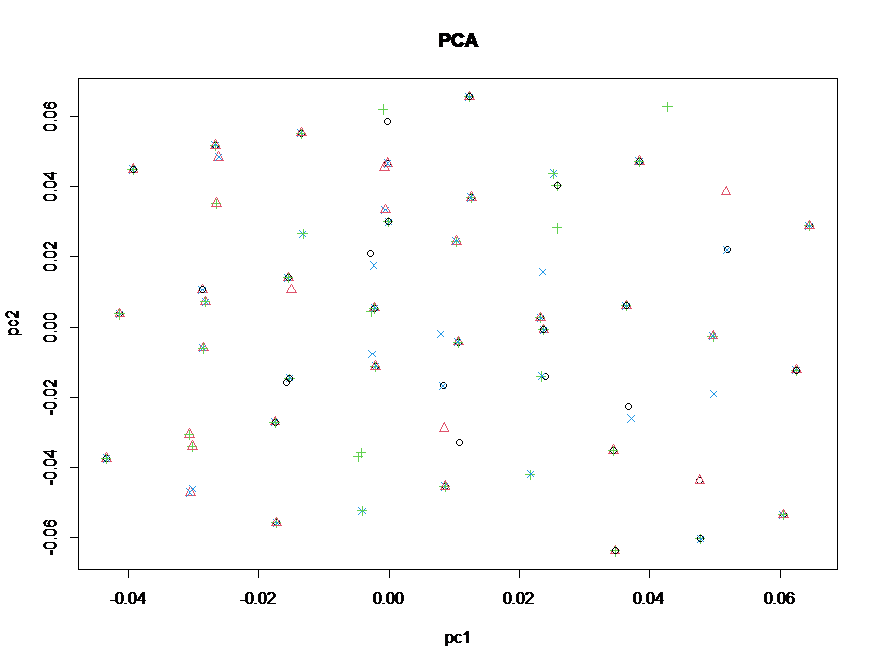

Supplement: Supplementary file 1 — Additional file 1: Figure 1 Principal component analysis based on genotyping data of 1001 participants. The distance of each sample on the horizontal and vertical axes represents the similarity distance influenced by the principal component. The stronger the relevance of participants, the closer they are in PCA; the weaker the relevance of participants, the more scattered they are in PCA. [file 12920_2021_1000_MOESM1_ESM.tif]
